# Repair of degenerative mitral regurgitation: An update

**DOI:** 10.21542/gcsp.2019.4

**Published:** 2019-03-31

**Authors:** David Richens, Anas Boulemden, Henry Skinner

**Affiliations:** Trent Cardiac Centre, Nottingham University Hospitals, Nottingham, UK

## Introduction

The recent development of catheter-based therapies for structural valve disease, such as mitral-TAVI, MitraClip and left atrial appendage occlusion devices, makes a review of surgery for degenerative mitral valve disease timely. In this personal perspective we discuss the evolution of mitral valve repair, the core principles involved and the evidence base behind it through the lens of a single UK-based surgical team operating for a quarter century, illustrating the techniques, outcomes and some of the pitfalls of intervention.

## The principles of repair

**Figure 1.**
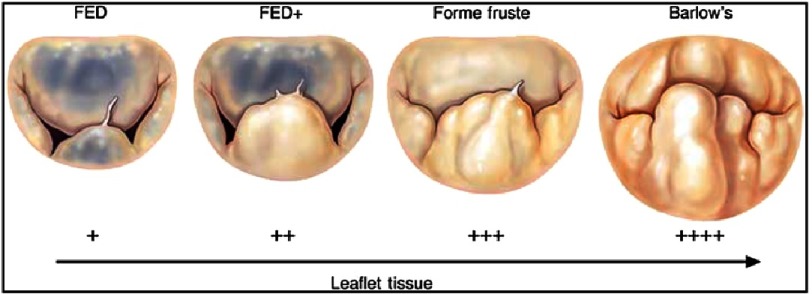
Spectrum of degenerative mitral valve disease, ranging from fibroelastic dysplasia (FED) to Barlow valve. (Adams DH, Rosenhek R, Falk V. Degenerative mitral valve regurgitation: best practice revolution. European Heart Journal. 2010;31(16):1958–1966).

Many different descriptions have been made attempting to classify degenerative mitral valve disease, ranging from fibroelastic deficiency to Barlow disease^1^ (Figure 1). Whichever system is employed, the important features are that as the mitral regurgitation gets worse, overload of the atrium and ventricle causes the annulus to change shape from that of a “D” on its side, with transverse diameter greater than vertical diameter, to an oval shape with vertical diameter greater than transverse. The annulus also becomes larger, the increase in diameter arising from stretching of the muscular attachment of the posterior leaflet (comprising 2/3rd of the annulus) rather than the fibrous attachment of the anterior leaflet (comprising 1/3rd annulus). Another important feature in advanced pathology is that there can be excess leaflet tissue, with prolapse of multiple scallops involving both leaflets and also calcification of the annulus and subvalvular structures.

Successful surgery is usually simple surgery and the core principles of repair are simple. There are only three. Restore the annular geometry, restore normal leaflet motion and create a large surface area of coaptation. Each of these factors should be respected and to achieve all three entails a detailed knowledge of the anatomy, function and pathology of the valvular structures.

BOX 1 Principles of repair

1. Restoration of annular geometry
2. Restoration of normal leaflet motion
3. Creation of a large surface area of coaptation

Whilst attempting to achieve these three primary goals, naturally the surgeon needs to keep the patient alive and ensure that left ventricular function is preserved. The end result of the repair must be a competent valve with no gradient to inflow (mitral stenosis), or outflow (systolic anterior motion) of the left ventricle.

## Evolution of mitral repair surgery

Mitral valve repair is certainly not new. Cutler and Levine reported a successful closed valvotomy in 1923^2^. Their single operation was performed through a median sternotomy but subsequently Souttar described the left thoracotomy approach in 1925^3^. For the next 40 years, mitral intervention largely consisted of closed valvotomy for rheumatic disease via a left thoracotomy.

**Figure 2.**
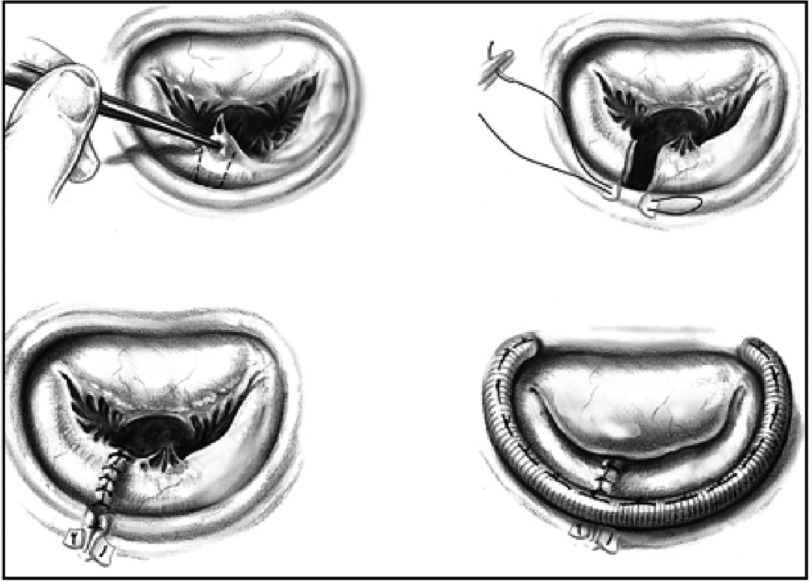
Mitral annuloplasty (From the milestone “French correction” paper). P2 quadrangular resection with ring annuloplasty.

By the late sixties, cardiac surgeons were routinely operating through a median sternotomy and employing cardiopulmonary bypass enabling direct vision of the mitral valve in the empty, motionless heart. Working in Paris, Carpentier began to demonstrate the benefits of repair over replacement^4^ in non-rheumatic pathology. He described a number of reproducible and successful techniques in such cases particularly annuloplasty and quadrangular resection (Figure 2).

**Figure 3.**
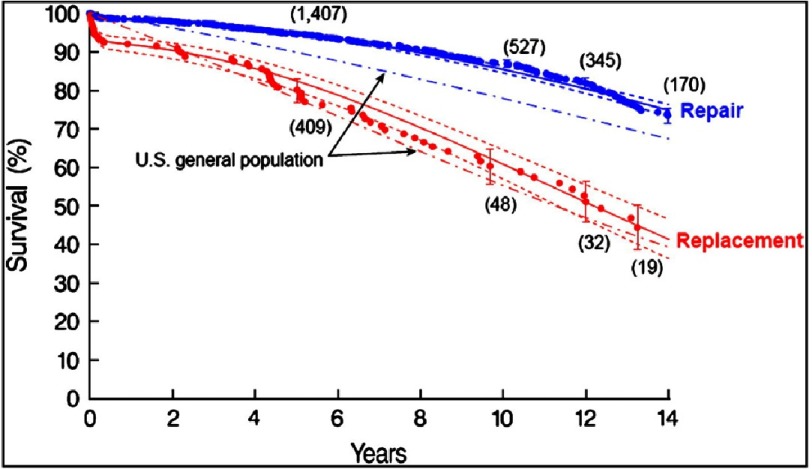
Unadjusted survival after mitral valve repair (*blue*) or replacement (*red*) compared with age and sex-matched US population (*dot-dash curves*). Each symbol represents a death, and vertical bars are 68% actuarial confidence limits. Numbers in parentheses represent patients remaining at risk. Solid lines are parametric survival estimates enclosed within dashed 68% confidence limits. (Gillinov AM, Blackstone EH, Nowicki ER, Slisatkorn W, Al-Dossari G, Johnston DR et al. Valve repair versus valve replacement for degenrative mitral valve disease J Thorac Cardiovasc Surg 2008; 135:885–893).

Eventually it became widely accepted that by using Carpentier’s techniques, repair rather than replacement was the procedure of choice for degenerative disease. An enduring survival benefit for repair over replacement was shown in a landmark paper by Gillinov^5^ in 2008, as shown in Figure 3.

**Figure 4.**
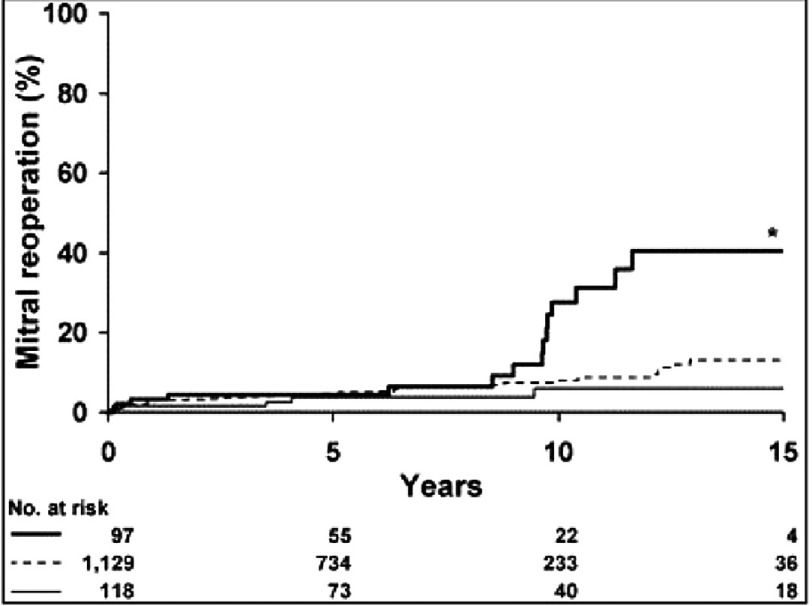
Probability of reoperation (mitral specific) after mitral valve repair versus replacement (biological or mechanical prosthesis). Zero time on abscissa represents date of operation and numbers at the bottom of the figure represent patients at risk. (Heavy solid line, biological prosthesis; HR, 2.4; *, *p* = 0.0016; light solid line, mechanical prosthesis; HR, 0.7; broken line, repair; HR, hazard ratio for reoperation compared). (Suri RM, Schaff HV, Dearani JA, Sundt TM, Daly RC, Mullany CJ et al. Survival Advantage and Improved Durability of Mitral Repair for Leaflet Prolapse Subsets in the Current Era. The Annals of Thoracic Surgery 2006 82, 819–826).

This improved outcome is now known to extend beyond short-term postoperative survival. Post operative stroke, long term survival and freedom from reoperation after repair are at least as comparable as that after mechanical replacement and statistically better than after tissue valve replacement (Figure 4)^6^.

**Figure 5.**
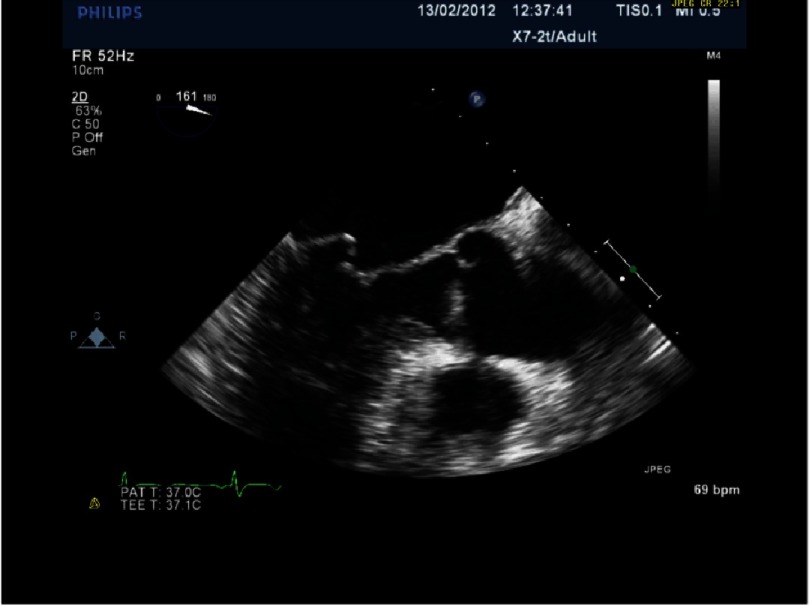
Severe MR with P2 prolapse.

**Figure 6.**
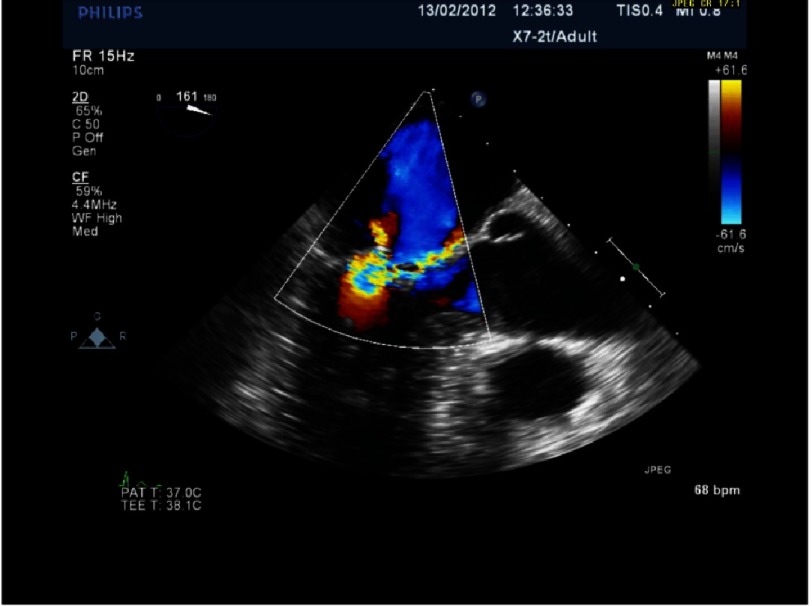
Severe MR with P2 prolapse and anteriorly directed jet (same patient as Figure 5).

**Figure 7A.**
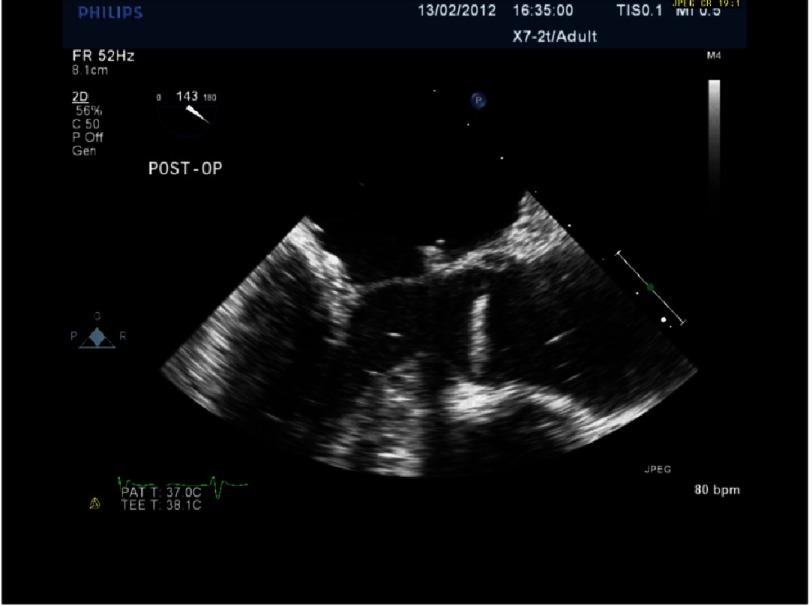
Postoperative repair of patient in Figures 5 and 6 after quadrangular resection and annuloplasty. There is now good coaptation.

**Figure 7B.**
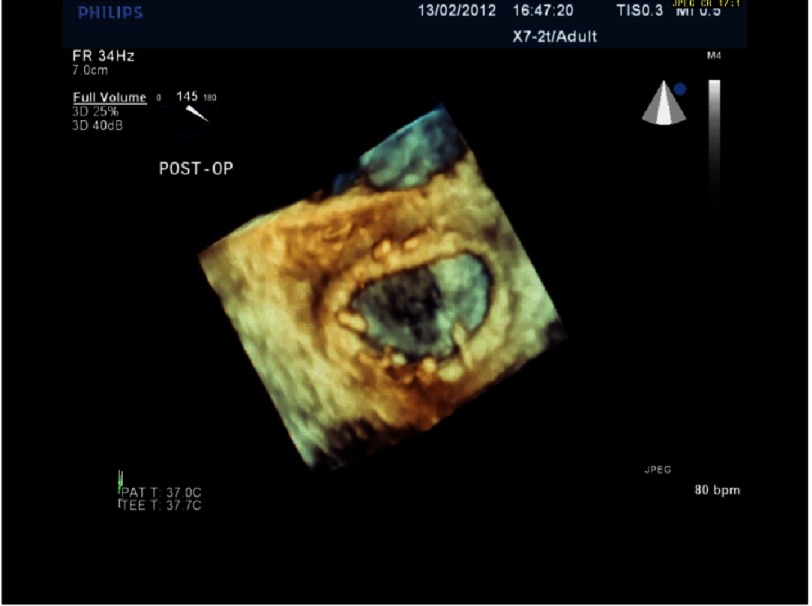
3D echo image of the same patient as in Figure 7A, post-repair.

## Case study 1

*Initially, we employed the classic mitral repair techniques described by Carpentier. This patient had severe mitral regurgitation due to P2 prolapse (*Figures 5 and 6*). The patient had a quadrangular resection of the posterior leaflet with a ring annuloplasty. The prolapsing segment of the P2 scallop was excised, the posterior leaflet then repaired by re-approximating the two remaining portions of P2 and the annular size reduced with an annuloplasty ring. Postoperative result was satisfactory as shown by*Figures 7A and 7B*.*

We have learned that whatever repair is performed, without an annuloplasty ring the repair is unlikely to stand the test of time. A full ring, as compared to a partial band, stabilizes the annulus and became our preference over time. The reason for this can be demonstrated by considering a cardboard box with a lid. Once the lid is removed it is possible to easily distort the box, replacing the lid gives the box structural integrity and reduces deformation when it is stressed. This is why a soft-top car suffers “scuttle shake” whilst a saloon version of the same model does not.

**Figure 8.**
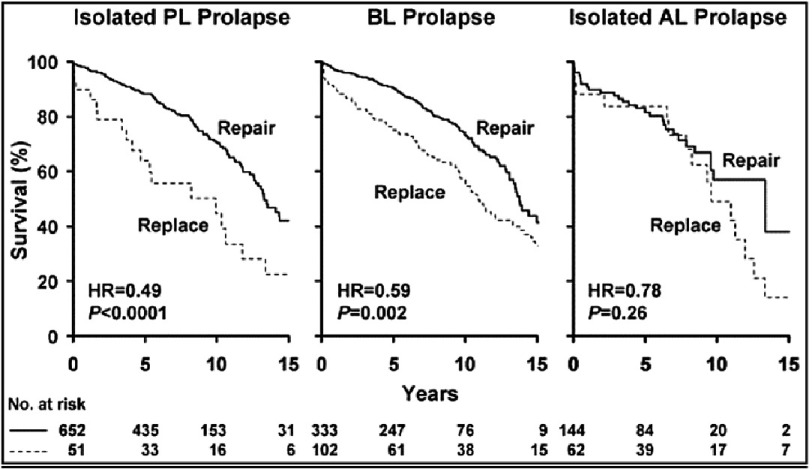
Probability of survival (death from any cause) among patients having mitral valve repair versus replacement divided into leaflet prolapse groups. Zero time on abscissa represents time of surgery and numbers at the bottom indicate patients at risk. (solid line, repair; broken line, replacement; AL, anterior leaflet; BL, bileaflet; HR, hazard ratio for survival of replacement group compared with repair group; PL, posterior leaflet). (Suri RM, Schaff HV, Dearani JA, Sundt TM, Daly RC, Mullany CJ et al. Survival Advantage and Improved Durability of Mitral Repair for Leaflet Prolapse Subsets in the Current Era. The Annals of Thoracic Surgery 2006 82, 819-826).

Although Carpentier’s techniques for isolated prolapse of the posterior leaflet have yielded satisfactory results, the results for more extensive disease involving both leaflets have not been as successful, as shown in Figure 8^7^. The search was on for alternative approaches, particularly for advanced disease.

**Figure 9.**
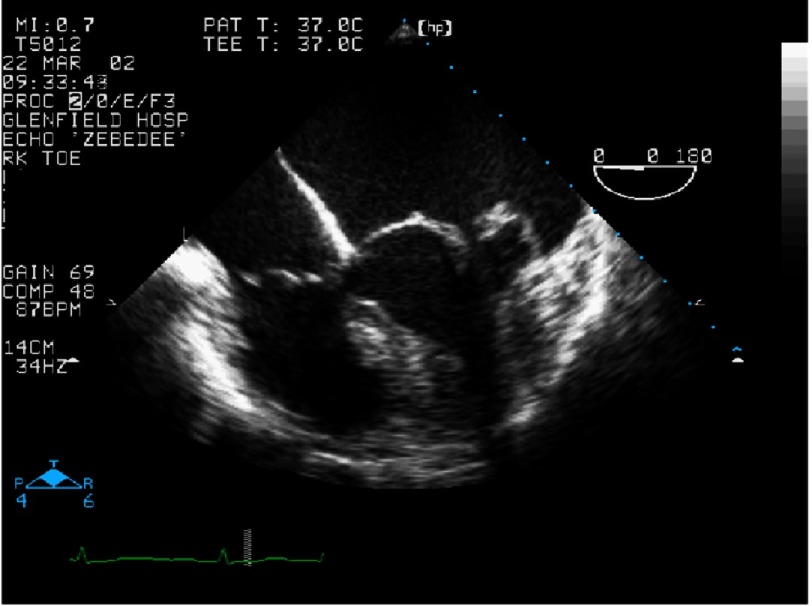
Barlow’s disease with thickened leaflets and bileaflet prolapse.

**Figure 10.**
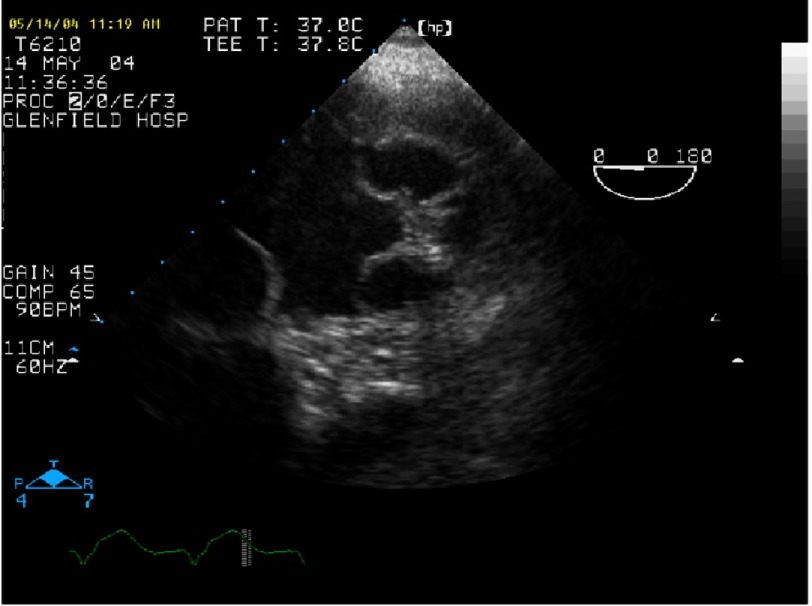
Short axis view of mitral valve showing the tip of A2 sutured onto the tip of P2 with orifices on either side (Same patient as in Figure 9).

## Case study 2 (Year 2008)

*This is a patient with bileaflet prolapse and annular dilatation due to extensive Barlow’s disease (*Figure 9*). The Alfieri suture offered an alternative to the Carpentier techniques. The anterior and posterior leaflets are sutured together along the line of coaptation at the region where the prolapse exists. This results in a double orifice inlet (*Figure 10*).*

An annuloplasty ring is needed in the “edge-to-edge” repair described by Alfieri, without which it has been demonstrated that the long-term results are poor^8^. Even when combined with annuloplasty, a competent Alfieri repair only satisfies two of the three previously stated principles; namely restoration of annular geometry and creation of a good area of coaptation. Leaflet motion is certainly not normal after surgery with the double orifice valve.

The quest for a more anatomic and enduring repair continued but by 2012 Worldwide results had reached the point where International Guidelines were published recommending liberalization of the triggers for referral for surgery^9^. These now included referral for mitral valve operation in severe regurgitation in *asymptomatic* patients, providing the likelihood of repair was high, rather than treatment by replacement of the valve.

In order to satisfy these guidelines and to be able to appropriately council patients it then became necessary to be able to predict the chances of successful repair. Lancelotti predicted the likelihood of a successful repair using echocardiography, depending on three factors; the Carpentier classification of the mitral regurgitation, the degree of mitral calcification and the degree of annular dilatation^10^. He stated that the likelihood of repair could be classified into either feasible, difficult or unlikely. For example; localized prolapse of one leaflet should be repairable, more extensive disease could still be repaired provided there is limited calcification and limited annular dilation. Furthermore a classification of difficult would not mean impossible but this would require greater surgical judgement and a higher degree of technical proficiency. Repair is unlikely if there is multi segment prolapse across both leaflets, extensive calcification (particularly when this involves the anterolateral commissure) and/or severe annular dilation (Table 1).

**Table 1:** Probability of mitral valve repair based on echo findings.

Etiology	Dysfunction	Calcification	Annular dilatation	Repair?
Degenerative	II: Localized prolapse (P2 or A2)	No/Localized	Mild/Moderate	Feasible
Ischemic	I or IIIb	No	Moderate	Feasible
Barlow	II: Extensive prolapse	Localized (annulus)	Moderate	Difficult
Rheumatic	IIIa but pliable anterior leaflet	Localized	Moderate	Difficult
Severe Barlow	II: Extensive prolapse (≥3 scallops, anterior commissure)	Extensive	Severe	Unlikely
Rheumatic	IIIa but stiff anterior leaflet	Extensive	Moderate/Severe	Unlikely
Ischemic	IIIb but severe valvular deformation		No or Severe	Unlikely

Annular calcification is a major surgical pitfall in our experience, particularly when widespread with islands of calcium extending down into the left ventricular myocardium and very specifically, when it is located in the annulus at the anterolateral commissure. This is an extremely difficult surgical problem and disastrous disruption of the left ventricle/atrio-ventricular junction may result if the calcium is extensively debrided, leading to severe hemorrhage and likely death of the patient.

When repairing advanced Barlow disease, it may be possible to stop any valve leaking if the annulus is made small enough and all the leaflet tissue is positioned below the level of the annulus, however the consequence of so doing is that the new coaptation point is brought dangerously close to the intra-ventricular septum, with a risk of systolic anterior motion (SAM). Varghese^11^ has described the risk factors, as shown in Table 2, that if present preoperatively, increase the likelihood of SAM.

**Table 2:** Risk factors for SAM.

Coaptation-septal distance <25 mm,
Sharp aorto-mitral angle <120,
Posterior Mitral leaflet height >15 mm,
Small left ventricular (LV) cavity LVIDD <45 mm,
Basal septal thickness >15 mm.

In our contemporary practice, asymptomatic patients with severe regurgitation are referred for surgery when there are signs of LV dysfunction or remodelling, new atrial fibrillation or raised pulmonary artery pressure. We saw more asymptomatic patients without such risk factors being referred as the likelihood of repair increased in line with our experience (Table 3).

**Table 3:** Indications for mitral valve repair (ESC/EACTS guidelines 2012).

Class I	Class II
• Symptomatic severe MR • Asymptomatic severe MR and LVESD>45 mm or EF<60	• Symptomatic severe MR and severe LV dysfunction* • Asymptomatic severe MR and new AF or PAP>50 mm • Asymptomatic severe MR if repair is likely in centre of excellence

In our practice, patients with lesser degree of mitral regurgitation also have repair if they are having concurrent CABG or other open-heart surgery. In all cases the key is the ability to predict the chances of successful repair using echocardiography.

**Figure 11.**
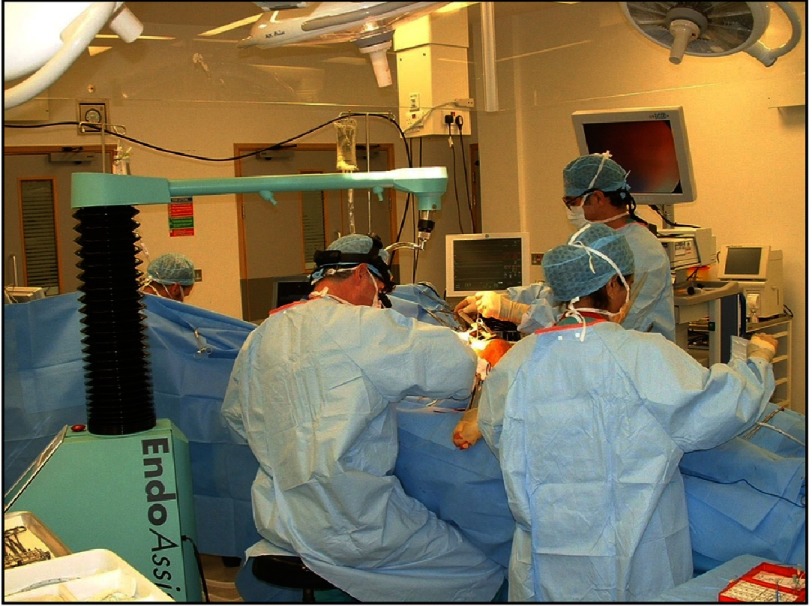
Trent Cardiac Centre operating theatre in 2000’s (minimal access mitral valve surgery) showing DR and HS plus Endo Assist robot.

## Alternative approaches to the mitral valve

By the year 2000 there were well developed endoscopic valve programmes throughout mainland Europe. Hugo Vanermen, working in Aalst, Belgium, had pioneered this approach with a series of elegant demonstrations of such surgery and published a number of landmark papers with excellent short and long-term outcomes for the technique. These can be summarized as improved cosmesis, reduced intra-operative trauma and enhanced recovery^12,13^. No such activity was taking place in the UK at that time, due to a combination of cost-pressures and the contemporary NHS regulatory oversight for introduction of new techniques.

Faced with a number of complex redo mitral procedures and recognizing the high risk associated with these operations when performed through a sternotomy, we decided to develop a minimally invasive mitral programme using an approach through a limited right thoracotomy thereby avoiding re-entry into the chest through the frozen anterior mediastinum. Figure 11 shows the team working in Nottingham performing endoscopic surgery via a limited port access approach on the right side and utilizing a single armed robot to hold and manipulate the endoscope.

The approach to the mitral valve, having started on the left side in the early years of surgery had migrated initially to the mid line and was now residing on the right side of the chest. Whatever the approach, the key principles of repair of the valve remained the same.

The unit performed 50 such cases^14,15^ and then stopped. We did not find the work cost effective as cases took longer operating times and the disposables were more costly. Although postoperative stay was reduced in our series, savings from this never met the increased cost of the disposables.

The industry drives much of the innovation in cardiac surgery and cardiology. Careful analysis of the efficiency and effectiveness of any new technology is required before mainstream acceptance of these devices and techniques. Surgeons, by their nature, want to try new strategies and devices and to be seen as innovators. We reappraised our endoscopic approach to the mitral valve following a case of severe regurgitation in a hemophiliac awaiting a liver transplant. This case had been turned down by other surgeons but was referred to us because of our experience with minimally invasive surgery. Despite full hematological back up, the patient died of massive postoperative bleeding.

The surgeon should be wary of hubris and also the lure of what is new, attractively presented to the surgeon by the industry. After reflection, we started doing less endoscopic work and returned instead to the study of the repair of the valve itself and tried to simplify repairs, rather than make them more complicated. The feeling was that we had concentrated more on the technology associated with minimal access (retractors, peripheral bypass, knot pushers etc.) rather than focusing onto the basic principles.

Subsequently, in order to obtain maximal visualization of the valve structures using a median sternotomy we routinely employed the superior, biatrial trans-septal approach as the cardiac incision of choice. This allows excellent access, full visualization without distortion of the heart, without any associated complication^16^.

**Figure 12.**
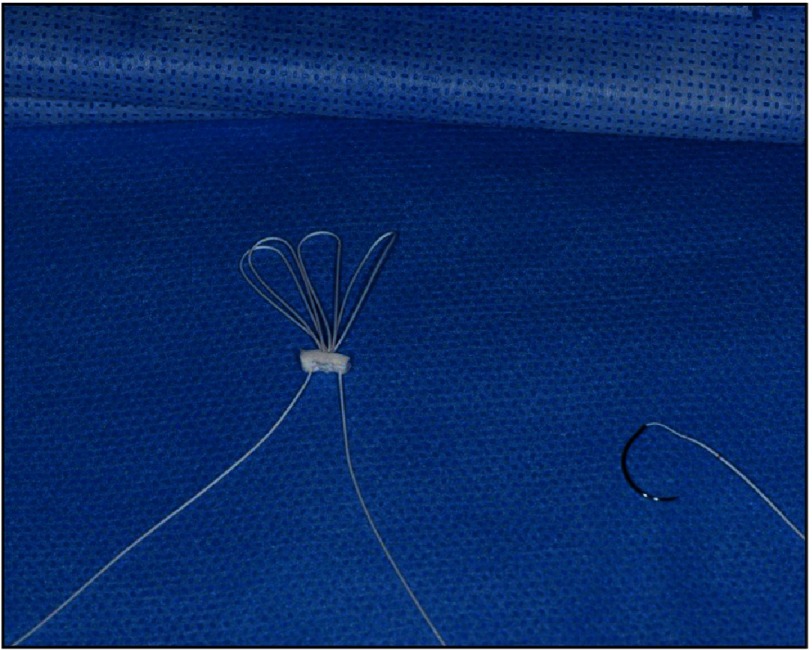
Artificial Gortex cords.

## Contemporary practice

From 2012 onwards, we started using chordal loops in repairs using neochordae made from PTFE (Figure 12). The loops come in pre-set lengths and the distance from the attachment point on the papillary muscle to the line of coaptation on the leaflet is measured using a sterile caliper at operation and this allows the surgeon to choose the appropriate size of loop.

**Figure 13.**
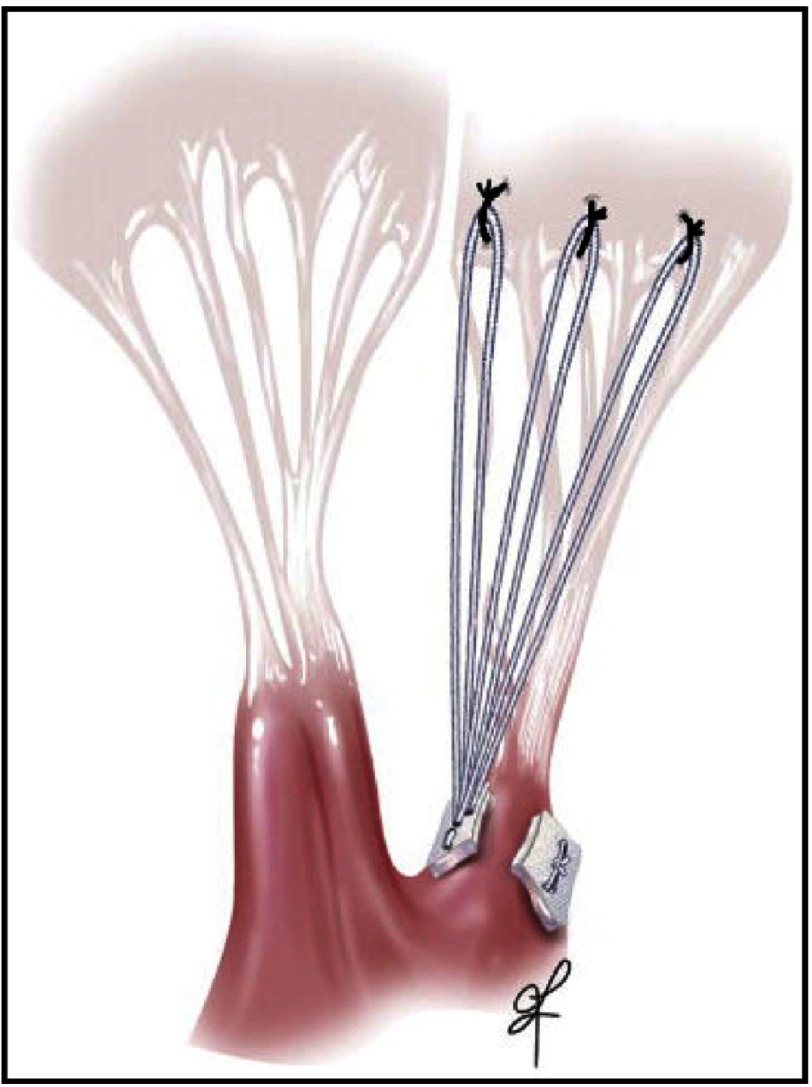
Mitral valve repair using artificial cords.

The approach is to suture the loops by pledget to the specific papillary muscle where the ruptured or elongated chord arise, then attach the apex of the loop to the atrial aspect of the corresponding leaflet scallop, at the level of the junction between the line of coaptation and the smooth inflow surface of the leaflet (Figure 13). These artificial chords should never cross the mid line of the leaflet or the transverse diameter of the valve (in other words they should respect the chordal geometry of the original valve). We found that by so doing, we could adjust the height of the leaflet coaptation by reattaching the loop higher or lower on the inflow surface of the leaflet as required. The coaptation point can be moved further away from the septum by so doing, using multiple loops higher on the posterior leaflet, thereby avoiding SAM. No leaflet tissue is resected.

## Case study 3 (2013)

**Figure 14.**
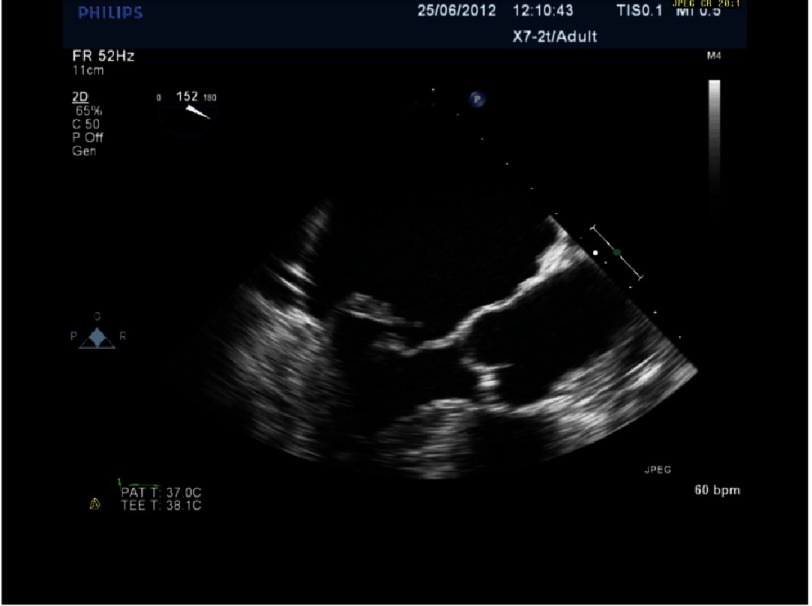
P2 prolapse due to ruptured cord.

**Figure 15.**
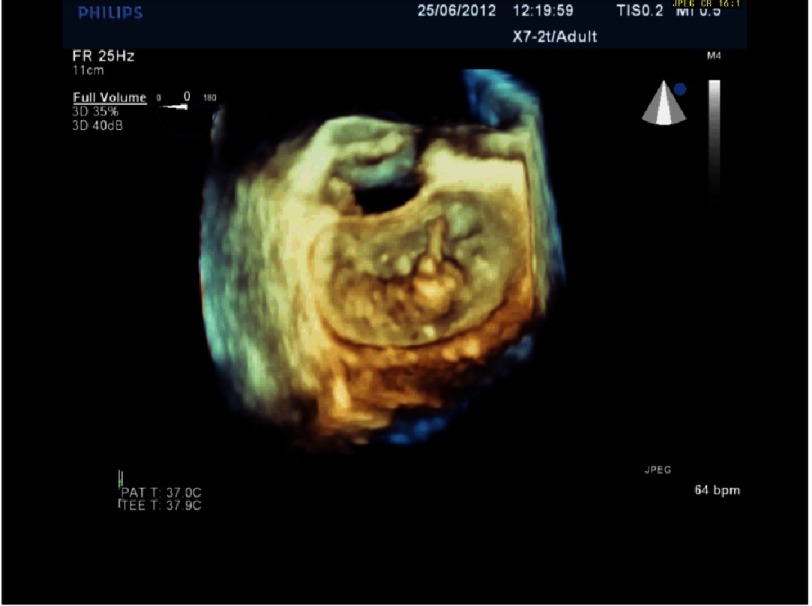
Same patient as Figure 14.

**Figure 16.**
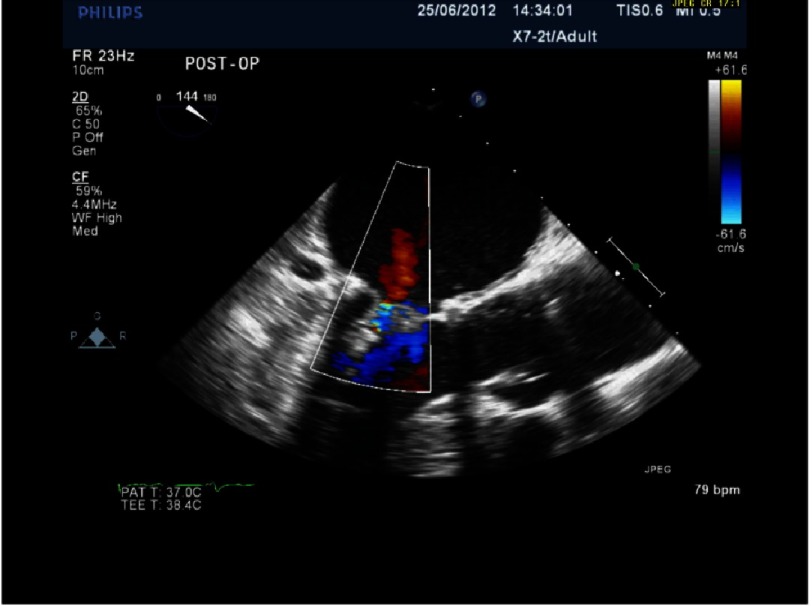
Same patient as 14, post-op repair with neochords and annuloplasty. There is now a good coaptation distance and no residual MR.

*A patient had P2 segment prolapse due ruptured cords, with severe mitral regurgitation (*Figures 14 and 15*). Instead of resection, P2 was retained and the ruptured cords replaced by artificial cords, a more physiological solution (*Figure 16*.)*

## Case study 4 (2015)

**Figure 17.**
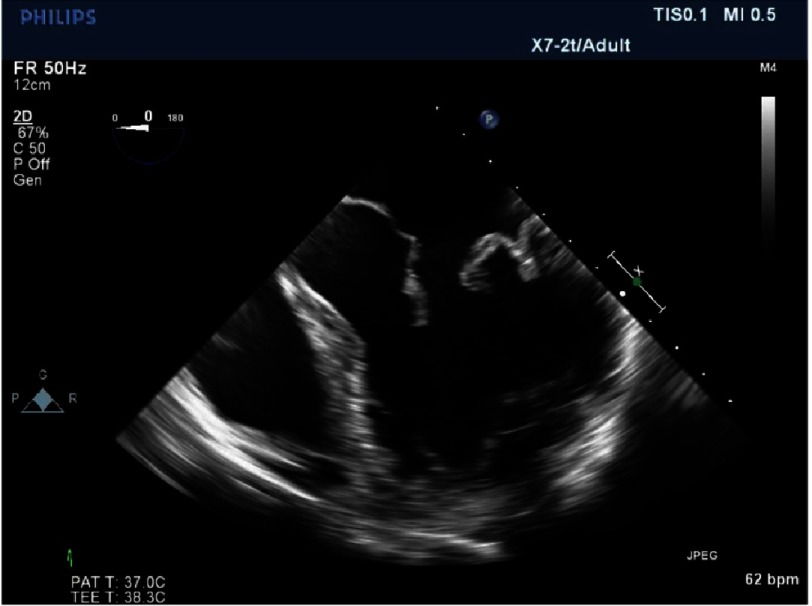
Patient with advanced Barlow.

**Figure 18.**
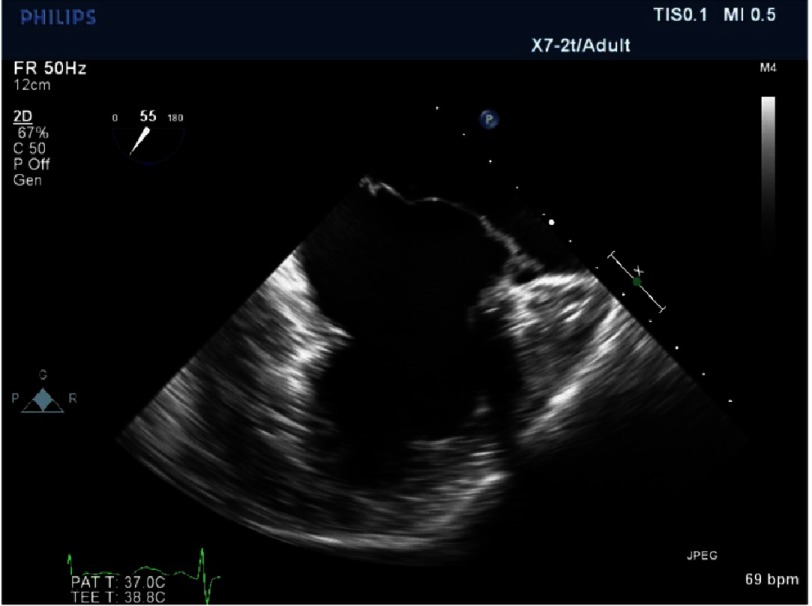
Same patient as 17. During systole, the leaflets billow above the annulus. In this patient there was prolapse of P1, P2 and A2.

*This is another patient but with advanced Barlow’s disease. In this instance three scallops are prolapsing: P1, P2 and A2 (*Figures 17 and 18*). There is excess tissue in both leaflets. The suitability for repair was open to question with bi-leaflet, multi-scallop prolapse and severe annular dilatation, however:-*

**Figure 19.**
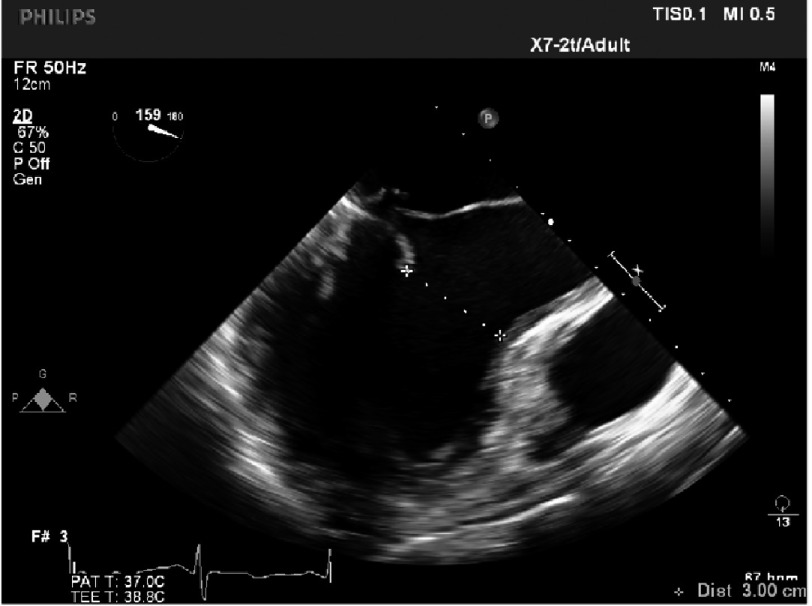
Distance between coaptation point and septum (>2.5 cm).

**Figure 20.**
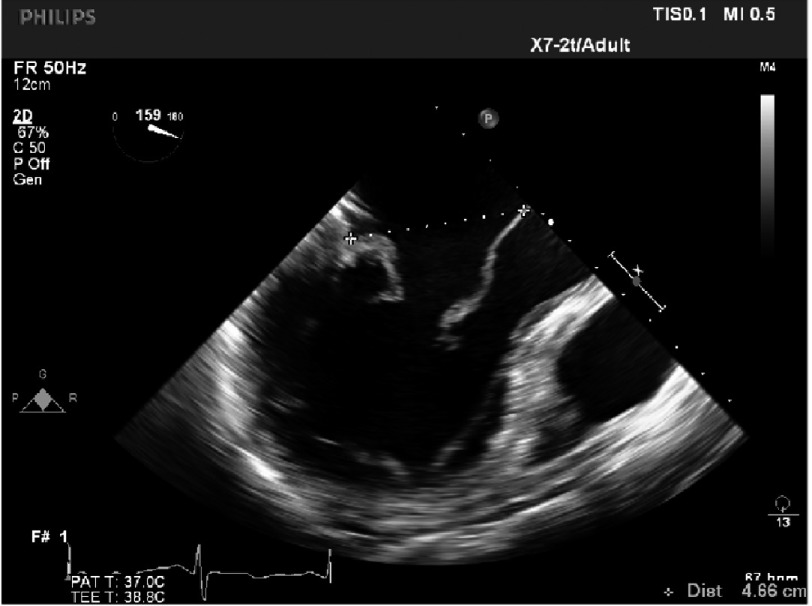
Dilated mitral annulus, adequate LV diameter and absence of septal hypertrophy.

**Figure 21.**
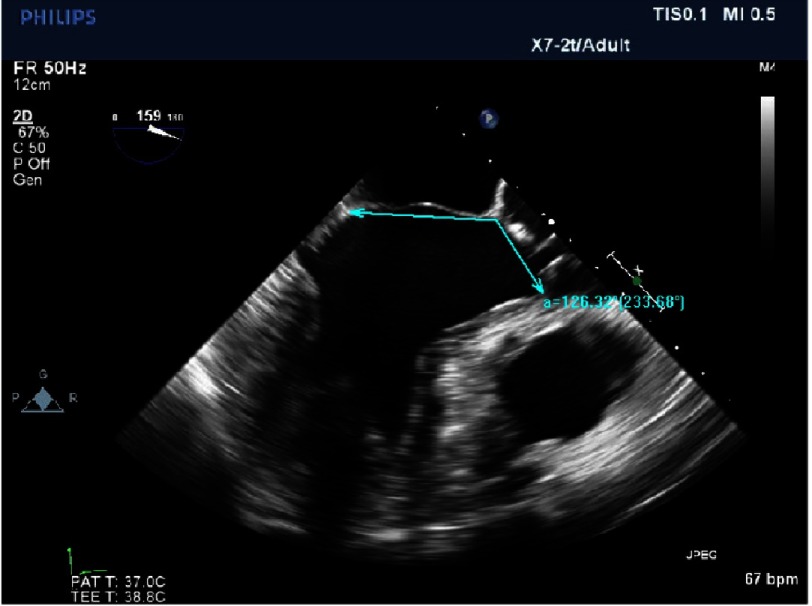
Aorto-mitral angle (>120).

• *There is a good distance between coaptation point and septum (*Figure 19*)*
• *The annulus is quite dilated (*Figure 20*) which potentially makes repair more difficult but, the LV cavity is not small and the septum not hypertrophied. The aorto-mitral angle is wide (Figure 21).*

**Figure 22.**
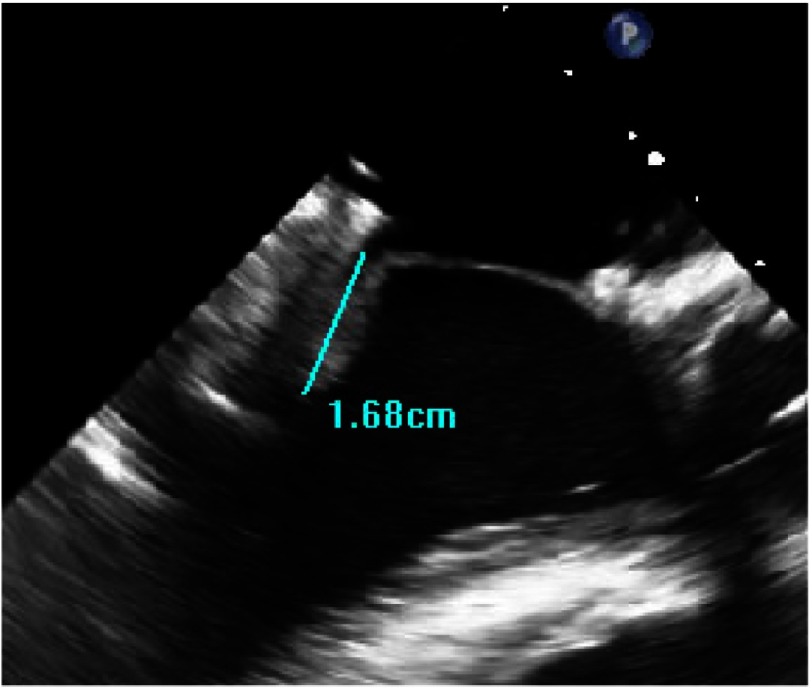
Post op result. Competent valve. Good Coaptation distance post-mitral valve repair and satisfactory C-Sept distance.

*This patient received a successful repair using chordal loops and annuloplasty ring. Note how much valvular tissue there is below the annulus (no tissue was resected). Nevertheless the new coaptation is well away from the septum without any SAM (*Figure 22*)*

The long coaptation length in particular protects against residual MR, we pursue a coaptation length of >5 mm

## Results using chordal loops

Over the three-year period 2013–16 we operated on 71 cases of degenerative mitral disease; 5 replacements were performed (all concomitant operations or redo’s of old repairs) and 66 repairs (93% repair rate of the whole series). There was one death (due to annular calcification) giving a mortality of 1.4%, and there have been no reoperations to date. First time operation for isolated degenerative mitral valve disease in this series had a 100% repair rate.

## Conclusions

The techniques of mitral repair are continuously evolving. The global trend is towards less invasive procedures. In the future, repair may well be performed by a variety of approaches using either surgery or by catheter based technology. Whatever method is chosen it is important to keep things as simple as possible and to adhere to first principles, namely the restoration of annular geometry, restoration of normal leaflet motion and creation of a good area of coaptation

There are dangers in intervention when there is extensive calcification. The clinician should be wary of hubris, the lure of what is new and the pressures from industry all of which may divert him or her away from these simple principles.
